# Future-Oriented Coping with Weather Stress among Mountain Hikers: Temperamental Personality Predictors and Profiles

**DOI:** 10.3390/bs11020015

**Published:** 2021-01-24

**Authors:** Piotr Próchniak, Agnieszka Próchniak

**Affiliations:** 1 Institute of Psycholoy, University of Szczecin, 71-017 Szczecin, Poland; 2 Department of Sociology, Pomeranian University, 76-200 Słupsk, Poland; agnieszka.prochniak@apsl.edu.pl

**Keywords:** coping with stress, weather, temperament, sensation seeking, personality, hikers, outdoor sports

## Abstract

The aim of the study was to explore temperamental personality traits as predictors of fu-ture-oriented coping with weather stress in a group of Polish mountain hikers. The subjects were 209 young mountain hikers (M = 21.20; SD = 3.70) who took three temperament–personality questionnaires, i.e., FCZ-KT Temperament Questionnaire, Sensation Seeking Scale IV and NEO-FFI- Personality Inventory, alongside a recently constructed scale for diagnosing future-oriented coping with weather stress in outdoor context, Preventive and Proactive Coping with Bad Weather Scale in Outdoor Sports. The regression analysis indicated that preventive coping with weather stress in hiking was predicted by activity, emotional reactivity, briskness, sensory sensitivity, experience seeking, agreeableness and conscientiousness. In turn, proactive coping with bad weather in hiking was predicted by endurance, activity, thrill and adventure seeking and extraversion. In turn, the cluster analysis revealed three distinct clusters of hikers characterized by diverse re-sults on the scales of preventive and proactive dealing with adverse weather, namely, *prudent hikers* (high preventive coping/high proactive coping), *reckless hikers* (low pre-ventive coping/high proactive coping) and *wary hikers* (high preventive coping/low proactive coping). The hikers in these clusters differed in terms of temperamental per-sonality traits.

## 1. Introduction

The word “hiking” is not precise. In North America, hiking is a vigorous walk on trails and in the countryside. In contrast, in Great Britain the term hiking is often used for all forms of walking. In this context, trekking in the Alps and a walk in the forest are both forms of hiking [1]. In Scandinavia, hiking is defined as a recreation which consists of walks (from less than an hour up to many days) in different landscapes, often in rural areas (p. 173) [2]. Sometimes hiking is used interchangeably with the words trekking, hillwalking, strolling or bushwalking.

Researchers distinguish different types of hiking, e.g., nordic walking (hiking with specially designed walking poles), dog hiking, glacier hiking or thru-hiking. One of the most popular types of hiking is mountain hiking. Mountain hiking, in the context of this paper, is understood as the activity of going for long walks in mountainous areas with altitude differences. This form of activity is gaining in popularity. The positive benefits of mountain hiking are multifaceted, including improving well-being, learning and improving skills, feeling healthy or deeply experiencing nature. Mountain hiking is easier than other forms of recreation in mountains (e.g., mountaineering), but it can still be challenging [3,4,5,6].

Weather is one of the most universal aspects of mountain hiking [7,8,9,10]. During day trips in the mountains, hikers might experience all kinds of weather, such as rapid variations of temperature in short time periods, strong winds, heavy rain, fog, dust or large amounts of mud. These weather conditions can be associated with significant mental and physical strain on hikers [11,12,13,14,15]. From a psychological perspective, hiking in harsh weather can change decision-making, perception, learning processes, anticipation of future events and can generate aggression or fear [16,17,18,19]. In turn, physiological changes can affect blood pressure, body temperature and the hormonal system or cause headaches and breathing problems [20,21,22]. The consequences of terrible weather can be frostbite and hypothermia [23,24,25].

Severe atmospheric conditions can create challenging situations in mountain hiking. This means that severe weather can create highly stressful situations and mountain hikers can experience weather stress. As such, the question of how hikers cope in these circumstances is an intriguing one [26]. 

Coping has a long tradition in source literature [27,28,29,30]. Lazarus and Folkman defined coping as the specific efforts, both behavioral and psychological, which people make in order to reduce or minimize stressful events, including unfavorable weather [31]. They distinguished two basic forms of coping, namely, problem-focused coping and emotion-focused coping. Problem-focused coping is aimed at “managing or altering the problem causing the distress.” In turn, emotion-focused coping is aimed at “regulating emotional responses to the problem” [32], p.150. Endler and Parker proposed a third basic form of coping, i.e., avoidance-oriented coping. Avoidance coping is aimed at minimizing, denying or even avoiding dealing with the stressor [33]. 

The forms of coping with stress mentioned thus far refer to present or past situations [34]. Schwarzer, on the other hand, distinguishes two forms of future-oriented coping: preventive coping and proactive coping [35]. Preventive coping is defined as undertaking actions in the present time aimed at minimizing or even reducing to zero the likelihood of experiencing future stress which is perceived as a threat [36]. Proactive coping is also future-oriented but it is related to future challenges. In proactive coping, the individual accumulates cognitive and emotional resources which facilitate taking advantage of future opportunities [37,38].

Source literature provides some tools that adequately measure future constructs, such as the Future-Oriented Coping Inventory and the Preventive Coping Resources Inventory (PCRI) [39]. The Future-Oriented Coping Inventory was inspired by the Proactive Coping Inventory, originally developed by Greenglass and his co-workers. The scales describe participants’ potential behaviours and attitudes in response to future stressors (excluding weather stressors) [40]. In turn, the Preventive Coping Resources Inventory (PCRI) is an instrument designed to measure coping resources useful for prevention [41]. There is one instrument applicable to coping with the future in outdoor contexts, namely, Preventive and Proactive Coping in Outdoor Sports [42]. This scale consists of two subscales: preventive coping and proactive coping. The preventive coping subscale focuses on preventive behaviours of recreationists directed at taking steps to minimise the adverse effects of difficult atmospheric conditions. In turn, the proactive coping subscale focuses on treating bad weather conditions in outdoor recreation as a challenge. 

Studies suggest that coping with stress may vary according to temperament and personality [43,44]. According to Strelau, temperament performs the function of moderators with an influence on difficult situations where there are implications of possible physical strain or even death. This author distinguishes the following aspects of temperament: briskness (the tendency to react quickly, maintain a high pace of activity), perseveration (the tendency to repeat behaviours after the situation that elicited them has changed), sensory sensitivity (the tendency to respond to sensory stimulation, even when it is of negligible value), emotional reactivity (the tendency to have an intensive emotional relationship with reality), endurance (the tendency to sustain activity in spite of adversity) and activity (the tendency to expresses a passion for seeking out activities connected with physical effort and risk). Furthermore, past research suggests the importance of these traits in active coping during difficult and stressful situations [45,46].

Zuckerman’s theory posits that people who undertake risky outdoor recreational activities are sensation seekers [47]. Sensation seeking is also a temperamental trait. Zuckerman identifies four factors that are involved in sensation seeking: thrill and adventure seeking (desire to engage in activities or sports involving danger), experience seeking (desire for experience through the mind and senses, a nonconforming life style or travel), disinhibition (desire for social and sexual disinhibition) and boredom susceptibility (aversion to repetition and routine). Studies suggest that thrill and adventure seeking is linked particularly to engagement in mountain activities [48,49,50,51,52,53,54].

Sensation seeking relates to coping in natural environments. For example, experienced climbers with high scores on sensation seeking can effectively manage their fear, control risk and facilitate success in climbing [55,56,57,58,59]. Furthermore, experienced climbers use different coping strategies, such as self-help, religion, denial or technology [60].

Not only temperament and sensation seeking predict stress coping strategies. Coping strategies can be also predicted by means of personality traits, namely, openness to experience (preference for variety, aesthetic sensitivity, intellectual curiosity, active imagination) [61], conscientiousness (the tendency to be organised, responsible or hard working) [61], extraversion (high levels of energy, activity or positive affect) [61], agreeableness (the individual’s tendency to develop altruistic, cooperative, modest or warm relationships) [62] and neuroticism (the tendency to be in a negative emotional state). Neuroticism correlates to emotional coping strategies and, in turn, active coping tends toward extraversion, as do conscientiousness and openness to experience. Agreeableness correlates to social support [62]. Preventive coping correlates with agreeableness, conscientiousness and openness to experience [63,64]. 

The goal of this study was to examine how personality-temperament traits predicted future-oriented coping with bad weather in hikers’ groups. Based on previous research, it was hypothesized that, in the context of bad weather conditions, proactive strategies used by hikers would correlate with activity, endurance, sensation seeking and extraversion. It was also posited that preventive coping under such conditions would, in turn, correlate with emotional reactivity, neuroticism and agreeableness and conscientiousness.

## 2. Method

### 2.1. Participants 

The sample consisted of 209 hikers (M = 21.20; SD = 3.70) (96 women (46%) and 113 men (54%)) who visited Polish mountains. 

The respondents practised hiking in the following Polish mountains ranges: the Tatras (66%), the Beskids (76%) and the Sudetes (48%). The sum of percentages is higher than 100 because respondents practiced hiking in more than one mountain range. The hikers practiced hiking in the summer (100%), in the spring (47%) and in the autumn (32%). Only 12% of respondents had personal experiences in winter hiking. They hiked in Tatras Mountain using mountain ice axes, hooks, ropes or harnesses. The average length of experience of hiking was 5.25 years (SD = 3.10).

### 2.2. Procedure 

The authors contacted mountain hikers through mountain tourism organisations, hiking associations, nordic walking clubs, etc. The leaders from these organisations, associations or clubs informed hikers about our research program. Mountain hikers who were interested in participating in the research clicked on a survey link. The hikers filled in questionnaires and sent them back to the authors. Participation in the research was voluntary. No incentive was provided for participation in the study.

All the participants were selected on the basis of the following criteria:

(a) Aged between 18 and 25 years old (in this age group, many people are involved in adventure recreation without professional knowledge, proper skills or information about risks) [65,66,67];
(b) Hiking in the mountains at least seven days per year;
(c) Involvement in hiking at in least one of three Polish mountain ranges, i.e., the Tatras, the Beskids or the Sudetes.

The Tatras have an alpine character (the highest peak is Gerlach, 2655 m above sea level). The characteristic feature of the Tatras climate is rapid weather variation in short time periods. There is a large amount of rainfall in the Tatra mountains for some peaks (about 1800 mm per year). In some places, snow remains all year round. The inherent element of the Tatras climate is represented by heavy winds. There is also a large risk of avalanches in the Tatra mountains in winter [68].

The Beskids are quite low mountains, below 2000 m. The main risk in the Beskids is rapid weather variation in short time periods, including unexpected rainfall and storms. There is a lot of mud on the trails. The highest peak in the Beskids is Babia Góra (1725 m above sea level), which is sometimes nicknamed *Mother of Bad Weather* [68].

The Sudetes are also quite low mountains, below 2000 meters. The area of the Sudetes stands out from the others due to the highest occurrence of mists in Poland (e.g., on Śnieżka, the highest peak in the Sudetes at 1602 m above sea level, there is an average of 306 foggy days per year and also a large number of cloudy days, 178 days per year on average). The Sudetes are characterized by warm, dry and gusty winds [68].

From 265 interested participants, 56 did not meet the eligibility criteria. All subjects gave their informed consent for inclusion before they participated in the study.

### 2.3. Measures

#### 2.3.1. Preventive and Proactive Coping with Bad Weather in Outdoor Sports

Preventive and Proactive Coping with Bad Weather in Outdoor Sports is 14-item tool [42]. The coefficient alpha reliabilities were 0.81 for the preventive scale, and 0.80 for the proactive scale. 

The preventive scale describes whether recreationists check the weather forecast before undertaking outdoor activity or follow the advice of weather services when they suggest caution in connection with the worsening weather. In turn, the proactive scale measures whether recreationists continue activity even when it is raining or cold or they like struggling with heavy winds, mud or fog during outdoor recreation. 

#### 2.3.2. FCZ-KT Temperament Questionnaire

The Regulative Theory of Temperament proposed by Strelau provides the theoretical framework for the FCT-KT. The FCZ-KT temperament questionnaire measure consists of 120 items. The items are grouped into six scales: briskness, perseveration, sensory sensitivity, emotional reactivity, endurance, and activity. Coefficients alpha reliability in the Polish version for the FCZ-KT were (in five independent studies) briskness (*Cronbach’s* α = 0.77–0.79), perseveration (*Cronbach’s* α = 0.79–0.81), sensory sensitivity (*Cronbach’s* α = 0.72–0.78), emotional reactivity (*Cronbach’s* α = 0.82–0.87), endurance (*Cronbach’s* α = 0.85–0.88) and activity (*Cronbach’s* α = 0.82–0.84) [69].

#### 2.3.3. Sensation Seeking Scale IV

*The Sensation Seeking Scale* IV is a tool to assess individual differences in optimal levels of stimulation. The Polish version of the SSS IV consists of 68 items comprising six subscales: thrill and adventure seeking (*Cronbach’s α* = 0.79), experience seeking (*Cronbach’s α* = 0.75), disinhibition (*Cronbach’s α* = 0.73), boredom susceptibility (*Cronbach’s α* = 0.70), the intellectual stimulation requirement (*Cronbach’s α* = 0.75) and general sensation seeking (*Cronbach’s α* = 0.82) [70]. In the current study, only the first four scales were used.

#### 2.3.4. NEO-FFI Personality Questionnaire

The *NEO-FFI* *Personality Questionnaire* is 60-item questionnaire that examines the five domains of personality: openness to experience (O), conscientiousness (C), extraversion (E), agreeableness (A) and neuroticism (N). (OCEAN).

Coefficients alpha reliability for the NEO-FFI in Polish sample were: openness to experience (*Cronbach’s* α = 0.68), conscientiousness (*Cronbach’s α* = 0.82), extraversion (*Cronbach’s* α = 0.77), agreeableness (*Cronbach’s* α = 0.68) and neuroticism (*Cronbach’s* α = 0.80) [71].

## 3. Results

Table 1 and Table 2 present the regression correlations between the structures of temperament, sensation seeking and personality traits for future-oriented coping with bad weather in outdoor sports. Regression analysis is a tool to establish a relationship between two variables. One of these variables is called the independent variable (predictor). The other variable is called the dependent variable, whose value is derived from the independent variable [72]. The structures of temperament, sensation seeking and personality traits were treated as independent variables and future-oriented coping strategies (preventive and proactive) as dependent ones. 

Preventive coping is predicted by emotional reactivity, activity (negatively), briskness, sensory sensitivity, experience seeking (negatively), agreeableness and conscientiousness.

Proactive coping is predicted positively by endurance, activity, thrill and adventure seeking and extraversion.

In the next stage of the study, cluster analysis was used in order to extract the basic profiles for individuals’ future-oriented coping strategies with bad weather in outdoor sports. The cluster analysis allows for the grouping respondents into specific clusters. The respondents in a cluster should be similar to one another and different from the respondents in other clusters [73]. The goal of the current cluster analysis was to extract the basic clusters for hikers who use preventive and proactive strategies in harsh weather, i.e., we were looking for hikers who had similar scores on the Preventive and Proactive subscales within a cluster and had different scores from the hikers grouped in other clusters.

We tested for different numbers of clusters. The K-means Cluster Method showed that the cluster model with the best fit was the three cluster model. In this model, variance between groups of hikers was higher than variance within groups for the Proactive and Preventive subscales simultaneously (higher variance between groups than variance within any single group is an important criterion in extracting clusters); see Table 3.

**Figure 1:**
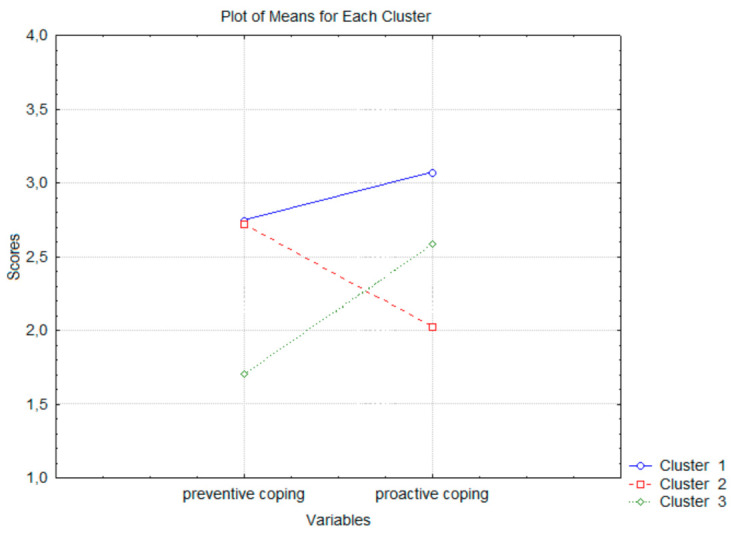
Clusters of future-oriented coping with weather stress in hikers; results of cluster analysis.

The cluster analysis revealed three profiles. The first contained the 69 hikers who had high scores on both coping scales (preventive and proactive). The second was composed of the 82 hikers who received high scores on the preventive coping scale and low on the proactive one. The third encompassed the 58 hikers with low scores for preventive coping and high scores for proactive coping. See Figure 1.

In the final step, we compared scores on temperament–personality variables in the three clusters of the hikers using one-way analysis of variance. One-way analysis of variance is a statistical technique to determine the existence of differences among several group means [74]; see Table 4.

The group of hikers using preventive and proactive coping strategies with bad weather in outdoor sports had higher scores for endurance, activity, briskness, thrill and adventure seeking and extraversion and lower scores on emotional reactivity, perseveration and agreeableness than the hikers who prepared to face the constraints of bad weather but did not use proactive coping strategies when practicing outdoor sports. 

The hikers who employed both proactive and preventive coping in outdoor recreation had also higher scores on briskness and sensory sensitivity, and lower scores on emotional reactivity, experience seeking, boredom susceptibility and conscientiousness in comparison to hikers who applied only proactive coping strategies with bad weather (without preventive coping strategies).

We also observed differences between hikers who used proactive coping and did not apply prevention and hikers who conversely used coping strategies with bad weather in practicing outdoor recreation (high preventive coping and low proactive coping).

Those who use proactive coping without preventing coping with bad weather in outdoor recreation had higher scores on endurance, activity, thrill and adventure seeking and experience seeking, and lower scores on emotional reactivity, sensory sensitivity and agreeableness in comparison to hikers who applied preventive coping strategies without proactive coping.

## 4. Discussion

The goal of this study was to examine how personality–temperament traits predicted future-oriented coping with bad weather in hikers. Two forms of future oriented coping with weather stress were correlated with multidimensional temperamental personality scales in the study.

The study produced numerous correlations between the structure of temperament (emotional reactivity, briskness, sensory sensitivity, endurance and activity) and coping with weather in hiking. 

Emotional reactivity positively correlated with preventive coping. This result suggests that preventive coping can minimize potentially negative emotions connected with bad weather conditions. The hypothesis in this regard was thus confirmed. Interestingly, briskness was demonstrated as positively correlating with preventive coping. The relationship between briskness and preventive coping seems reasonable. Bad weather constraints are dynamic and unpredictable and anyone facing these constraints must therefore be capable of responding to a changing situation if they wish to survive in natural environments. 

Why does sensory sensitivity positively correlate with preventive coping? Severe weather is highly intensive stimuli. It is thus no surprise that a individuals results high on this variable will respond well to a potential deterioration in atmospheric conditions by employing preventive coping. However, it must also be remembered that the sensory sensitivity scale is characterized by relatively low reliability and, as such, the results must be interpreted with some caution.

Endurance and activity positively correlated with proactive coping. The hypothesis in this regard was thus confirmed. The positive relationship between proactive coping with harsh weather conditions and endurance is clearly reasonable. For hikers, severe weather is often a source of disagreeable emotions or even suffering. It would therefore only be people with considerable physical strength, pain resistance and perseverance who would not shy away from tough weather conditions but would consider them in terms of confrontation. In other words, hikers scoring high on endurance are biologically predisposed toward confronting severe weather. 

The correlation between the activity trait and proactive coping with weather stress in hiking seems to be equally clear and explicable. Harsh atmospheric conditions present a high-risk situation. This situation is thus a challenge and, as such, attractive to individuals with a strong need for stimulation. Interestingly, high intensity activity correlated negatively with preventive coping. Perhaps hikers with a high propensity to take risks believe so strongly in their own skills that they see no need to protect themselves against the possible negative consequences of severe weather conditions. It is probable that they attach little or no importance to the risks posed by such conditions.

The strategies for coping with weather stress in the hikers correlated with level of sensation seeking. Thrill and adventure seeking positively correlated with proactive coping, as previously assumed. The interpretation of this dependence was similar to the previous analysis of the relationship between activity and proactive coping with weather stress in hiking. It seems that confrontation of bad weather conditions responds to what matters to those who appreciate the issues grouped under the thrill and adventure seeking subtrait [75]. Interestingly, experience seeking correlated negatively with preventive coping with bad weather in practising hiking. Severe atmospheric conditions are potentially highly dangerous. It is thus no surprise that a hiker scoring low on experience seeking will respond well both to a potential deterioration in weather conditions by employing preventive coping and when it comes to direct confrontation with harsh weather.

The analysis consisted of the possible correlations between five personality traits and future- oriented coping with bad weather conditions in hiking. Extraversion predicted proactive coping, thus confirming the hypothesis. Confrontation with strong wind, heavy rain, snow or low and high temperatures requires high levels of power and energy. Given this fact, the positive relationship between extraversion and proactive coping is clear, understandable and easy to predict.

The research did not confirm the links between neuroticism and preventive coping. In turn, agreeableness and conscientiousness predicted preventive coping in the hikers. Given this fact, agreeable hikers most often take prognoses of atmospheric conditions seriously and made decisions about action in the mountains based on weather forecasts. In turn, conscientious people are goal-directed and future-oriented and analyze risks associated with their projects or tasks. In consequence, they take protective measures against these risks. In this context, conscientious hikers make protective efforts connected with the weather hazards. 

The cluster analysis indicated three groups of hikers differing in the scales of proactive and preventive coping. The first profile contained the individuals who had high scores on both coping scales (*prudent* *hikers*). The second was composed of the hikers who scored low on the proactive coping scale and high on the preventive one (*wary* *hikers*). The third profile encompassed the individuals who with high scores for proactive coping and low scores for preventive coping (*reckless* *hikers*).

The profiles of the three groups of hikers differing in their proactive and preventive coping with bad weather in hiking are outlined below.

### 4.1. Prudent Hikers

Hikers in this type are aware of the risks posed by bad weather conditions and are careful in such situations. They tend to focus on the potential negative consequences of remaining in a threatening weather situation (higher preventive coping). Like reckless hikers, they seek thrill and adventure in natural surroundings but, in contrast to reckless adventure seekers, they have higher levels of energy to cope with bad weather in outdoor recreation (high extraversion) and can persistently continue hiking in spite of adverse weather (high endurance). 

In comparison with reckless hikers, prudent hikers are more organised and persistent in situations of weather stress than reckless participants (high conscientiousness). Thus, they treat forecasts pertaining to possible changes in the weather seriously and may have fewer accidents in the wilderness. 

### 4.2. Reckless Hikers 

The characteristics for this type are a need for stimulation (high activity and sensation seeking). Reckless hikers thus maintain a positive attitude toward new events which demand risk-taking, perceiving them as a source of pleasant stimulation. They enjoy effort and physical activity and their values are those involving an exciting life. Hikers in this type get bored quickly (low boredom susceptibility).

Reckless hikers probably are little aware of the risks posed by bad weather conditions. They tend not to focus on the potential negative consequences of remaining in a threatening weather situation. Reckless hikers do not take weather forecasts seriously and fail to heed the warnings and recommendations of the meteorological experts (little agreeableness). They do not exactly analyze the dangers associated with their goals or try to take protective measures against these weather risks (little conscientiousness). They do not take warnings seriously and may therefore have more accidents than other types of hikers. 

### 4.3. Wary Hikers

The fundamental psychological feature of hikers in this group is that they will try to prepare for bad weather but if they come into contact with an adverse situation, lack adequate resources for confronting it effectively. Strength and a tendency to take risks do not constitute their strengths (low endurance, low sensation seeking). In comparison to other types, they probably display a lack of consistency in their implementation of operations in adverse conditions in mountains which, in the case of this study, are those of severe weather. 

Hikers of this type have high preventive coping scores. They are aware both of future weather dangers and of the negative consequences of failing to prepare for bad weather. Hikers in this type take weather forecasts seriously. When deciding on a holiday destination, they take the risks that may arise into consideration. They probably like hiking (occasionally soft and easy hiking) in good weather conditions.

### 4.4. Limitations of the Study and Future Directions 

First, we must remember that the relationship between personality–temperament traits and future-oriented coping does not mean cause and effect, especially when correlation values are low. Therefore, the idea of linking between these variables must be handled with great care.

An important limitation of the present study is that the respondents were all young people. This fact limits the generalizability of results. In future research, it would be important to assess not only young people but other groups of adults. In this study, the variable of gender was not controlled for. Future research should also take the gender variable into account.

In future research, it would be interesting to analyze the relationship between psychological variables and the future-oriented coping scale among individuals practicing other outdoor sports (e.g., kayakers, sailors, etc.). The current research focused only on hikers. 

In this study, only some of the temperament–personality traits, which may well be of significance to future-oriented coping in bad weather, were subjected to analysis. This means that the current research was conducted from the perspective of psychobiological characteristics. Future research should consider variables with more cognitive character, such as personal values. Values have more cognitive character than temperamental personality traits as they describe what is important for people while at the same time playing a motivational role regarding people’s chosen activities, similar to personality traits. Temperament and personality traits tell us what people like, while values tell us what is important to them. 

## 5. Conclusions 

This article presented theoretical analyses and empirical research of temperament–personality predictors of future-oriented coping with bad weather in mountain activity. Contrary to mainstream research into the influence of weather on behaviour, our research attempted to show how any given outdoor hiker deals with weather adversities in the context of a wide spectrum of psychobiological variables. It turns out that hikers use different ways of coping and dealing with dangerous weather phenomena depending on multidimensional psychological variables, such as temperament and personality traits.

It seems that the results of the study can supplement the knowledge about the psychobiological basis of the functioning of individuals in the natural environment. Moreover, the results may also inspire further research on the relationship between the individual and the natural environment. In turn, from a practical point of view, instructors of outdoor adventure can use these results for diagnosis when they begin working with clients.

Threatening weather conditions are an inherent element of human cognitive, emotional and behavioural functioning. One could go so far as to state the weather constitutes a hidden dimension of personal behaviour. The attitude one has toward weather (especially if it poses a direct threat to health or even life) says a lot not just about the weather but about the person, i.e., their personality, motives, coping, what is important in their life and what they are driven by in life, including their hopes and fears. 

## Figures and Tables

**Table 1:** Preventive coping with weather stress in hiking and temperamental personality predictors; the results of multiple linear regression.

Variables	Structure of Temperament		
	β	t(202)	*p*
Emotional Reactivity	0.18	2.39	0.01
Endurance	−0.06	−0.85	n.s.
Activity	−0.24	−3.57	0.01
Briskness	0.19	2.71	0.01
Sensory Sensitivity	0.27	4.03	0.01
Perseveration	−0.12	−1.69	n.s.
R^2^ = 0.24; F(6, 202) = 10.80; *p* < 0.01			
	**Sensation Seeking**		
	**β**	**t(204)**	***p***
Thrill and Adventure Seeking	0.05	0.83	n.s.
Experience Seeking	−0.24	−3.21	0.01
Disinhibition	0.00	−0.09	n.s.
Boredom Susceptibility	−0.07	−1.02	n.s.
R^2^ = 0.09; F(4, 204) = 5.05; *p* < 0.01			
	**Traits of Personality**		
	**β**	**t(203)**	***p***
Extraversion	0.00	0.11	n.s.
Neuroticism	0.06	0.93	n.s.
Agreeableness	0.21	3.17	0.01
Conscientiousness	0.23	3.37	0.01
Openness to Experience	−0.02	−0.42	n.s.
R^2^ = 0.11; F(5, 203) = 5.52; *p* < 0.01			

**Table 2:** Proactive coping with weather stress in hiking and temperamental personality predictors; the results of multiple linear regression.

Variables	Structure of Temperament		
	β	t(202)	*p*
Emotional Reactivity	−0.12	−1.70	n.s.
Endurance	0.30	4.24	0.01
Activity	0.33	5.22	0.01
Briskness	0.03	0.46	n.s.
Sensory Sensitivity	0.06	0.97	n.s.
Perseveration	0.01	0.01	n.s.
R^2^ = 0.34; F(6, 202) = 18.10; *p* < 0.01			
	**Sensation Seeking**		
	**β**	**t(204)**	**p**
Thrill and Adventure Seeking	0.27	3.85	0.01
Experience Seeking	0.02	0.26	n.s.
Disinhibition	−0.01	−0.15	n.s.
Boredom Susceptibility	−0.06	−0.83	n.s.
R^2^ = 0.07; F(4, 204) = 4.31; *p* < 0.01			
	**Traits of Personality**		
	**β**	**t(203)**	***p***
Extraversion	0.19	2.60	0.01
Neuroticism	0.02	0.27	n.s.
Agreeableness	−0.08	−1.14	n.s.
Conscientiousness	0.06	0.87	n.s.
Openness to Experience	0.08	1.19	n.s.
R^2^ = 0.06; F(5, 203) = 2.71; *p* < 0.05			

**Table 3:** Variance within and between groups for preventive and proactive coping with weather stress in hikers; the results of clustering analysis.

Model	Variable	Variance between Group	df	Variance within Group	df	F	*p*
Two Clusters	Preventive coping Proactive coping	44.60 11.25	11	37.82 62.04	207 207	244.07 37.54	0.01 0.01
Three Clusters	Preventive coping Proactive coping	44.62 41.69	22	37.80 31.60	206 206	135.88 121.60	0.01 0.01

**Table 4:** The structure of temperament, sensation seeking and personality traits for three profiles of future-oriented coping with weather stress in hikers; results of one-way analysis of variance.

Temperament-Personality Variables	Coping						F
	Preventive High Proactive High (a)		Preventive High Proactive Low (b)		Preventive Low Proactive High (c)		
	M	SD	M	SD	M	SD	
Emotional Reactivity	2.25	0.64	2.63	0.63	2.42	0.59	6.82 a–b **, a–c *, b–c **
Endurance	3.00	0.53	2.53	0.45	2.77	0.51	16.5 4 a–b **, b–c **
Activity	2.87	0.54	2.53	0.45	2.92	0.57	11.9 3 a–b **, b–c **
Briskness	3.25	0.44	3.02	0.37	3.02	0.42	7.36 a–b **, a–c **,
Sensory Sensitivity	3.08	0.51	2.98	0.50	2.70	0.55	9.00 a–c **, b–c **
Perseveration	2.79	0.50	2.97	0.51	2.88	0.44	2.68 a–b *,
Thrill and Adventure Seeking	7.57	2.32	6.48	2.25	6.98	2.25	4.42 a–b **, b–c **
Experience Seeking	5.27	1.80	5.15	2.01	6.17	1.93	5.27 a–c **, b–c **
Disinhibition	5.91	2.44	5.95	2.11	6.12	2.20	0.14
Boredom Susceptibility	3.46	1.65	3.93	1.93	4.12	1.92	2.21 a–c *,
Extraversion	6.95	1.98	6.10	1.82	6.06	2.21	4.34 a–b **, a–c *,
Neuroticism	4.28	1.87	4.23	1.84	4.56	2.10	0.56
Agreeableness	5.24	2.08	5.86	1.88	4.79	2.30	4.71 a–b *, b–c **
Conscientiousness	7.05	1.98	6.59	2.18	5.89	2.21	4.72 a–c **
Openness to Experience	4.31	2.08	3.93	1.60	4.55	2.29	1.72

* *p* < 0.05; ** *p* < 0.01.

## Data Availability

Data is available on request to the corresponding author (P.P.).
